# Clinical predictors of a positive genetic test in hypertrophic cardiomyopathy in the Brazilian population

**DOI:** 10.1186/1471-2261-14-36

**Published:** 2014-03-13

**Authors:** Julia Daher Carneiro Marsiglia, Flávia Laghi Credidio, Théo Gremen Mimary de Oliveira, Rafael Ferreira Reis, Murillo de Oliveira Antunes, Aloir Queiroz de Araujo, Rodrigo Pinto Pedrosa, João Marcos Bemfica Barbosa-Ferreira, Charles Mady, José Eduardo Krieger, Edmundo Arteaga-Fernandez, Alexandre Costa Pereira

**Affiliations:** 1 Laboratory of Genetics and Molecular Cardiology, Heart Institute (InCor), University of São Paulo, São Paulo, Brazil; 2 Clinical Unit of Cardiomyopathies, Heart Institute (InCor), University of São Paulo, São Paulo, Brazil; 3 Federal University of Espírito Santo, Vitória, Brazil; 4 Chagas Disease and Heart Failure Outpatient Service, PROCAPE-University of Pernambuco/UPE, Recife, Brazil; 5 Federal University of Amazonas, Manaus, Brazil

**Keywords:** Genetics, *MYH7*, *MYBPC3*, *TNNT2*, Molecular, Screening

## Abstract

**Background:**

Hypertrophic cardiomyopathy is a genetic autosomal dominant disease characterized by left ventricular hypertrophy. The molecular diagnosis is important but still expensive. This work aimed to find clinical predictors of a positive genetic test in a Brazilian tertiary centre cohort of index cases with HCM.

**Methods:**

In the study were included patients with HCM clinical diagnosis. For genotype x phenotype comparison we have evaluated echocardiographic, electrocardiographic, and nuclear magnetic resonance measures. All patients answered a questionnaire about familial history of HCM and/or sudden death. β-myosin heavy chain, myosin binding protein C, and troponin T genes were sequenced for genetic diagnosis.

**Results:**

The variables related to a higher probability of a positive genetic test were familial history of HCM, higher mean heart frequency, presence of NSVT and lower age. Probabilities of having a positive molecular genetic test were calculated from the final multivariate logistic regression model and were used to identify those with a higher probability of a positive molecular diagnosis.

**Conclusions:**

We developed an easy and fast screening method that takes into account only clinical data that can help to select the patients with a high probability of positive genetic results from molecular sequencing of Brazilian HCM patients.

## Background

Hypertrophic cardiomyopathy (HCM) is a genetic autosomal dominant disease characterized by left ventricular (LV) hypertrophy, without dilatation, usually asymmetric and mainly septal in the absence of any other cardiac or systemic disease that can lead to secondary hypertrophy [1,2]. The main symptoms, when present, are dyspnea on exercise, angina, heart palpitations, pre-syncope or syncope, but many patients may remain asymptomatic and some may have sudden death (SD) as the first manifestation of the disease. The estimated prevalence is 0.2% (1:500), corresponding to 0.5% of all cardiomyopathies [3].

The disease is caused by a mutation in sarcomere, disc-Z, or calcium handling genes. So far, over 20 genes have been associated with the disease, and over 1,000 different mutations have been described. However, the most common genes causing the disease are β-myosin heavy chain (*MYH7*), myosin binding protein C (*MYBPC3*), and troponin T (*TNNT2*) [4].

Molecular diagnosis is very important for several reasons. When the clinical diagnosis is a certainty, establishment of the molecular defect is a diagnostic confirmation, because HCM is a genetic disease. On the other hand, a genetic diagnosis can help in uncertain cases, such as when this is little hypertrophy, hypertrophy in athletes, or hypertensive patients are being screened [2]. In addition, genetic diagnosis allows the identification of children and adults with subclinical manifestations of the disease, especially in the familial context.

Although genetic testing is important, and it is recommended by the European Cardiology Society, it is not yet a reality in most clinical settings, mainly due to its high cost and the lack of well-established genotypic and phenotypic correlations. Some authors previously reported the use of clinical features to predict a positive genetic test [5,6], as this can help to optimize genetic testing by prioritizing patients with a high chance of a positive genetic test.

Thus, this work aimed to find clinical predictors of a positive genetic test in a Brazilian tertiary centre cohort of index cases with HCM.

## Methods

### Patients

All the subjects included in the study are HCM index patients clinically diagnosed by expert cardiologists. All of them are patients treated in the respective hospitals the researchers are affiliated and were invited to participate of the research during the periodic consultation. The assistant physician explained about the research and referred them to the Molecular genetics Analysis Laboratory. A septum thickness above 15 mm in the absence of any other disease that could lead to secondary hypertrophy was the criterion used. It included patients from the Heart Institute, a tertiary centre at the University of São Paulo Medical School, but also patients from other cities in Brazil, namely Vitória, Manaus, and Recife. All participants signed the informed consent, and the University of São Paulo Hospital’s Research Ethics Committee (CAPPesq) approved the project. Only one patient per family was included in the present analysis.

### Examinations

For genotype x phenotype comparison, clinical data were obtained from the patients’ medical records. We have evaluated echocardiographic, electrocardiographic, and nuclear magnetic resonance measures, when available. All patients answered a questionnaire about familial history of HCM and sudden death. The presence of a familial history was divided in three categories: absent, when there was no history, present, when at least one relative had a confirmed diagnosis for HCM and unsure, when the patient mentions that there is a history of cardiac disease in the family but without an established HCM diagnosis.

### Electrocardiography

Tracings were analysed for the presence or absence of atrial fibrillation, LV hypertrophy, left bundle branch block, left atrial enlargement, delta waves and abnormal Q waves.

### Ambulatory ECG monitoring

Tracings were recorded with 3 bipolar leads for 24 hours using commercially available equipment and analysed by an experienced reader. Complex ventricular arrhythmia was defined as the presence of non-sustained ventricular tachycardia (NSVT) defined as >3 consecutive ventricular ectopic complexes occurring at a rate of >100 beats/min.

### Echocardiography

Two-dimensional echocardiography with M-mode recording was obtained following the recommendations of the American Society of Echocardiography (ASE) [7]. The resting systolic gradients were measured with colour-guided continuous-wave Doppler (CWD) across LV cavity and outflow tract, avoiding the effect of mitral regurgitation when present. The examinations were performed using standard equipment with 2.5 and 3.5 MHz transducers.

### Genetic testing

The polymerase chain reaction (PCR) was performed from DNA using primers that cover the entire coding region from β-myosin heavy chain (*MYH7*), myosin binding protein C (*MYBPC3*), and troponin T (*TNNT2*) genes (primer sequences available upon request). After PCR, the fragments were purified with the ExoSAP-IT® enzyme (GE Healthcare). The sequencing reaction was performed with BigDye Terminator V3.1 Cycle sequencing Kit® (Life Technologies) and EDTA/ethanol precipitation protocol. The samples were sequenced in an automatic sequencer ABI3500xl (Life Technologies).

The sequences were evaluated with the SeqMan program (DNASTAR Lasergene, Madison, WI) and compared with the reference sequence in the NCBI database. For *MYH7*, *MYBPC3,* and *TNNT2*, the references were, respectively, NM_000257.2, NM_000256.3, and NM_000364.2 [8]. When an undescribed mutation was found, we used bioinformatics algorithms to evaluate the pathogenic potential of the alteration. The SIFT [9] and *Polyphen*[10] programs were used only for substitutions, and the *MutationTaster*[11] was used to analyze deletions, insertions, and intron alterations. A mutation was labeled as pathogenic if (1) it had been previously described as causing disease; (2) it generated an aminoacid change and was considered pathogenic by all 3 programs above or in 2 programs but the aminoacid was conserved.

### Statistical analysis

Statistical analyses were performed with the SPSS program 15.0. The ANOVA test was used to compare means, Fisher’s exact test for frequencies comparison, and uni- and multivariate logistic regression for constructing the prediction model. The variables with a statistical difference in the ANOVA test were tested in the univariate logistic regression and independent variables with a p value < 0.1 were included in the multivariate logistic regression. We did not use any hierarchical or stepwise approach in the multivariate logistic regression model.

Accuracy of the final prediction model was explored through ROC analysis. Sensitivity, specificity, positive and negative predictive values according to model cut offs were determined. The prediction model was generated with the probability of a positive genetic test derived from the regression model (where (β) are the regression coefficients of variables in the final model). These probabilities were imputed in a ROC analysis and a cut-off maximizing specificity and sensitivity was chosen.

$$p=\frac{e^{\beta_{0}+\beta_{1^{x}}}}{1+e^{\beta_{0}+\beta_{1^{x}}}}$$

## Results

This study analysed 268 patients, 58% males and 42% females with a median age of 46 years (SD = 15.6). The youngest was 13 years old and the oldest 90. We obtained the data from ECG and echocardiogram from all the patients and data from holter and resonance were obtained respectively from 176 and 116 patients. The variable used for the analysis were obtained as followed: presence or absence of atrial fibrillation, obtained from ECG, septal and posterior wall thickness, left atrium size and ejection fraction, obtained from echocardiogram, medium heart rate and presence of NSVT from Holter and presence or absence of fibrosis from cardiac resonance.

The median septal thickness was 20 mm (SD = 6), posterior wall thickness (PW) was 12 mm (SD = 5), left atrium size (LA) was 42 mm (SD = 8), and ejection fraction (EF) was 71% (SD = 9). There were no statistical differences in these criteria regarding sex.

Among the patients, we found a pathogenic mutation in 131 of them (48.8%). Seventy-eight (59.5%) of the mutations were in the *MYH7* gene, 50 (38.2%) in the *MYBPC3* gene, and 3 (2.3%) in the *TNNT2* gene. All variant used for this analysis were considered pathogenic according to our criteria described on Methods section. The full variant list can be found a previous article from our group [12]. The comparison between clinical features and presence or absence of an identified mutation has shown that patients with a mutation are, on average, younger in age, younger in age at diagnosis, have higher average cardiac frequency, and higher frequency of patients with NSVT (Table 1).

**Table 1:** Clinical features with absence or presence of a mutation in one of the studied genes

**Mutation**							
	**Absence**			**Presence**			
	**Mean**	**SD**		**Mean**	**SD**		**p value**
**Age at diagnosis (years)**	38	16		33	13		**0.026**
**Current age (years)**	48	17		43	13		**0.028**
**MCF (bpm)**	67	9		71	11		**0.006**
**Septum (mm)**	20	6		21	5		0.179
**PW (mm)**	12	3		12	6		0.581
**LA (mm)**	42	8		43	8		0.164
**EF (%)**	71	9		71	9		0.638
		**Count**	**Frequency (%)**		**Count**	**Frequency (%)**	
**Sex**	M	79	57.2		76	58.3	0.902
	F	59	42.8		54	58.5	
**AF**	No	91	93.8		90	87.4	0.150
	Yes	6	6.2		13	12.6	
**Obstructive**	No	73	64.6		81	70.4	0.397
	Yes	40	54.1		34	29.6	
**NSVT**	No	63	75.9		50	54.9	**0.004**
	Yes	20	24.1		41	45.1	

*SD* Standard deviation, *MCF* medium cardiac frequency, *PW* posterior wall thickness, *LA* left atrium size, *EF* ejection fraction, *AF* atrial fibrillation, *obstructive* ventricular gradient above 30 mmHg, *NSVT* non-sustained ventricular tachycardia. p-values < 0.05 are marked in bold.

Familial history of HCM was also correlated with the presence or absence of an identified mutation (Table 2), and it was found that when there is a proven familial history, the chance of finding a mutation is significantly higher than when there is no familial history. When the familial history was unsure, as in the cases when the patient mentioned relatives with cardiac disease, but without a clinical diagnosis of HCM, the odds of identifying a mutation were higher than in those with a negative family history and statistical similarity to those with a confirmed familial history.

**Table 2:** Comparison of positive, negative and unsure HC familiar history and mutation identification

**HC familial history**							
		**Absent**		**Present**		**Unsure**	
		**Count**	**Frequency**	**Count**	**Frequency**	**Count**	**Frequency**
**Mutation**	Absent	16	69.6%	23	37.1%	33	50%
	Present	7	30.4%	35	62.9%	33	50%

**Figure 1:**
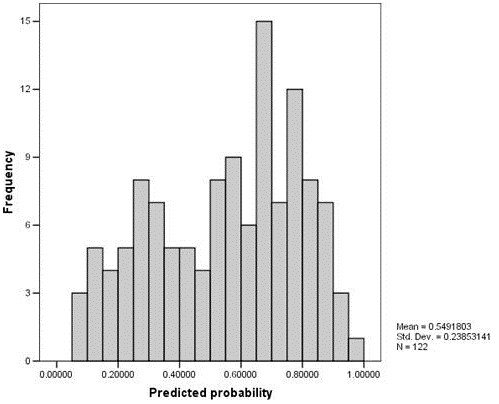
Predicted probability distribution.

**Figure 2:**
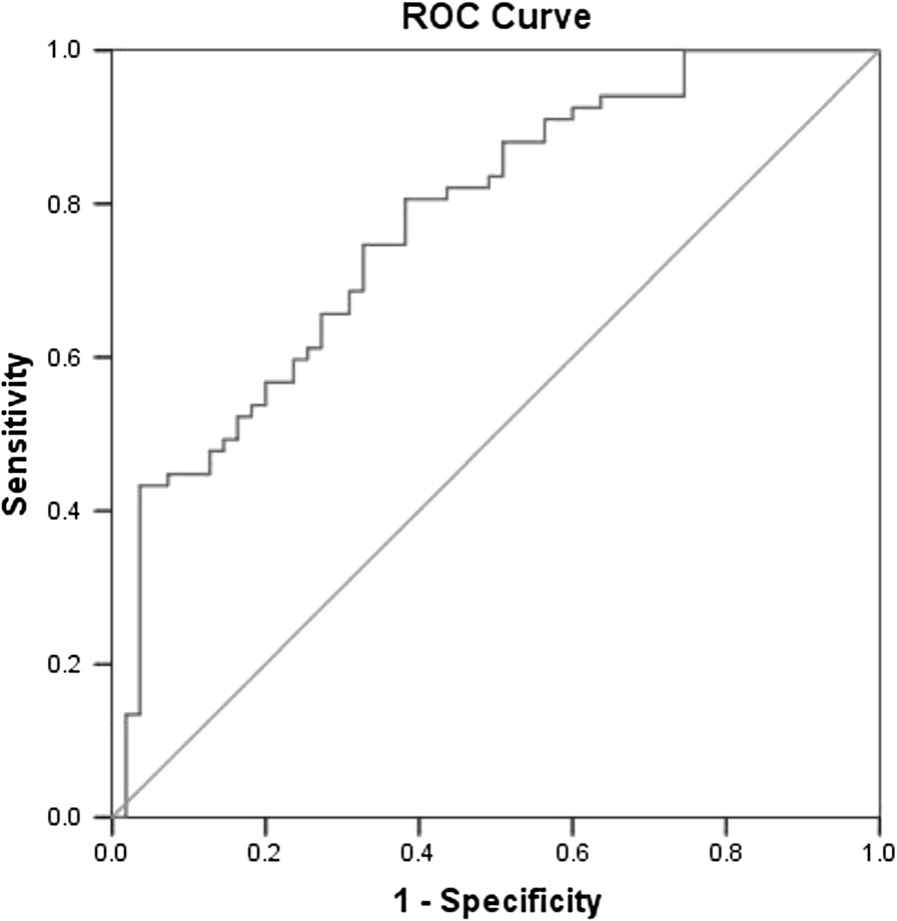
ROC curve for the developed model.

The variables with a statistical difference in the ANOVA test were tested in the univariate logistic regression and independent variables with a p value < 0.05 were included in the multivariate logistic regression: familial history of HCM, average heart frequency, NSTV and age (Table 3). Probabilities of having a positive molecular genetic test were calculated from the final multivariate logistic regression model and were used to identify those with a higher probability of a positive molecular diagnosis. The predicted probabilities distribution and ROC curve are shown respectively in Figures 1 and 2. The AUC is 0.775 and 0.55 was used as the cut-off value for the ROC curve.

**Table 3:** Variables included in the multivariate logistic regression

	**B**	**S.E.**	**Wald**	**df**	**Sig.**	**Exp(B)**	**95.0% C.I. for EXP(B)**	
							**Lower**	**Upper**
**HCFH**			7.222	2	0.027			
**HCFH (1)**	1.767	0.661	7.152	1	0.007	5.856	1.603	21.387
**HCFH (2)**	1.261	0.665	3.594	1	0.058	3.53	0.958	13.004
**AHF**	0.044	0.021	4.471	1	0.034	1.045	1.003	1.089
**NSVT**	1.603	0.479	11.193	1	0.001	4.969	1.943	12.709
**Age**	−0.053	0.018	9.211	1	0.002	0.948	0.916	0.981
**Constant**	−2.507	1.718	2.13	1	0.144	0.081		

*HCFH* Familial history of HC, *HCFH (1)* familial history of cardiac disease with confirmed HC diagnosis, *HCFH (2)* familial history of cardiac disease without HC diagnosis, *AHF* average heart frequency, *NSVT* non-sustained ventricular tachycardia.

## Discussion

### Genetic test positivity prediction model

The genetic test positivity prediction model developed in this study is useful to analyse *a priori* a patient’s chance of finding a mutation when ordering genetic testing. In the clinical practice scenario, cardiologists can give a more accurate estimate to patients regarding the molecular test. In the large scale screening scenario, such as in national cascade screening projects, only patients with a greater chance of having a positive genetic test would be analysed, which could optimize the use of reagents and analysis time. This model is particularly important in centres with fewer resources, because the genetic test is still very expensive. Especially in the Brazilian scenario, where few academic centres have the structure and budget to perform the test, this method can serve as a cost-effective tool. Our main focus with this model is the cascade screening. Patients with borderline hypertrophy or with uncertain diagnosis were not included in this study, so such predictor may not be accurate for this group.

The sensitivity and specificity values can be adjusted according to a particular interest. For example, if one wants to maximize the number of positive patients included, the sensitivity and specificity can be modulated by changing cut-off values. In the simulations for the studied population, adjusting the predicted probability cutoff for 0.34, which represents an approximate 90% sensitivity, from the 122 included in the analysis, 91 would be tested. Of those, 60 patients would be positive. From the 31 patients not tested, only 7 would have a positive result, so we would not be testing 24 patients predicted as negative. These savings, in a national screening program, can signify an important economic resource.

To applied this model, one can use the values from Table 3 to calculate the predicted probability of a positive genetic test. P is equal the predicted probability, βo is the constant value, β1 is the variable constant and x is the variable value if continuous or 0 and 1 if the variable is categorical.

In this work, we are using predicted probability, thus what we can conclude is regarding higher or lower probabilities, not certainties. For example, we saw in the logistic regression model that each patient’s year addition decreases the chance of finding a mutation in the genetic test. We did not define a cutoff for this measure, meaning that one can find the mutation in any age, but the older the patient is, the lower is the probability of finding a mutation. On the other hand, the presence of a confirmed HCM family history increases the chance of finding a mutation almost 6 times when compared to patients who don’t have a positive HCM family history.

Ingles et al. [5] also used this approach to identify positivity predictors for genetic testing in an Australian HCM population. The multivariate analysis of this population identified female sex, LV thickness, HCM familial history, and SD familial history as associated with a higher chance of mutation identification. The authors considered familial history as a key predictor of a positive genetic test in their population, with a 3 times higher chance of a positive result compared to patients without a familial history, which was similar to what we found in this study. In their study, this detection rate was even higher when the patient also had an SD familial history. Differently from what was found in this study, age at diagnosis was not significant in the Australian population, although the p value was 0.052.

Another study performed by Gruner et al. [6] in a Toronto HCM population showed that in these patients age at diagnosis, female sex, HCM familial history, and SD familial history were also correlated with a higher probability of a mutation identification. In addition, the study correlated hypertension and dyslipidaemia as negative predictive factors, with a higher frequency of both in genetically negative patients. Other strong predictors identified were morphology subtype, as previously described [13] and maximal wall LV thickness and LW thickness.

The identification of NSVT as one of the predictors for a positive genetic test is a very interesting observation, since this is one risk factor for sudden death in HCM patients. In a previous work, Olivotto et al. [14] compared patients with and without an identified sarcomeric mutation and found that there was a difference regarding those, with patients with a positive genetic test being related to a less favourable clinical outcome, especially regarding end stage heart failure, but not sudden death. This finding in our study may show that the presence of a mutation can indeed be related with a higher risk of sudden death related risk factors.

### Study limitations

The limitations of this study are the lack of patients with hypertension, since it was an exclusion criterion for HCM diagnosis in the participating centres; therefore, we could not test it as a negative predictor. Also, we only studied the three most important genes (*MYH7, MYBPC3,* and *TNNT2*), thus patients with mutations in other sarcomeric genes may be wrongly labelled as mutation negative. But the frequency of these genes in HCM is very low, so we believe that the lack of these data does not change the results found.

These finding may be limited to the Brazilian population. Replication studies in other populations should be made to confirm these results.

As new technology becomes available, such as next generation sequencing screening techniques, this reality may change and screening programs will have to constantly adapt to new molecular data.

## Conclusion

In conclusion, we developed an easy and fast screening method that takes into account only clinical data that can help to select the patients with a high probability of positive genetic results for molecular sequencing of Brazilian HCM patients. This method can be applied in centres with limited resources to save time and money.

## Competing interest

No competing financial interests exist. None of the authors had any funding by commercial agencies. Júlia Marsiglia, Flávia Laghi Credidio, Théo Gremen Mimary de Oliveira and Rafael Ferreira Reis had scholarships from national Science funding agencies (CNPq and CAPES). The other researchers are employees from the respective institutes and didn’t receive any extra funding for developing this work and writing or publishing this article.

## Authors’ contribution

JDCM - acquisition of data, analysis and interpretation of the data, Statistical analysis, Drafting of the manuscript. FLC - acquisition of data, analysis and interpretation of the data, critical revision of the manuscript for important intellectual content. TGMO - acquisition of data, analysis and interpretation of the data, critical revision of the manuscript for important intellectual content. RFR - acquisition of data, analysis and interpretation of the data, critical revision of the manuscript for important intellectual content. MOA - acquisition of data, critical revision of the manuscript for important intellectual content. AQA - acquisition of data, analysis and interpretation of the data, critical revision of the manuscript for important intellectual content. RPP - acquisition of data, analysis and interpretation of the data, critical revision of the manuscript for important intellectual content. JMBBF - acquisition of data, analysis and interpretation of the data, critical revision of the manuscript for important intellectual content. CM - acquisition of data, critical revision of the manuscript for important intellectual content. JEK - obtaining funding, critical revision of the manuscript for important intellectual content. EAF - conception and design of the research, acquisition of data, analysis and interpretation of the data, critical revision of the manuscript for important intellectual content. ACP - Conception and design of the research, analysis and interpretation of the data, statistical analysis, obtaining funding, critical revision of the manuscript for important intellectual content, Supervision. All authors read and approved the final manuscript.

## Pre-publication history

The pre-publication history for this paper can be accessed here:

http://www.biomedcentral.com/1471-2261/14/36/prepub
